# Supplementary material for: High-throughput targeted gene deletion in the model mushroom Schizophyllum commune using pre-assembled Cas9 ribonucleoproteins
Source: Sci Rep. 2019 May 21;9:7632. doi: 10.1038/s41598-019-44133-2 (PMC6529522; doi:10.1038/s41598-019-44133-2)
Supplement: Supplementary file 1 — Supplementary Material [file 41598_2019_44133_MOESM1_ESM.docx]

**Supplementary material**

**High-throughput targeted gene deletion in the model mushroom *Schizophyllum commune* using pre-assembled Cas9 ribonucleoproteins.**

**Peter Jan Vonk, Natalia Escobar, Han A. B. Wösten, Luis G. Lugones, Robin A. Ohm***

Microbiology, Department of Biology, Faculty of Science, Utrecht University, Utrecht, The Netherlands

Padualaan 8, 3584 CH Utrecht, The Netherlands

* Corresponding author: r.a.ohm@uu.nl

**Supplementary Table 1**. Summary of results across three replicates per condition. The average values and standard deviations are given.

| Strain | Cas9 RNP | Nourseothricin resistant transformants | Nourseothricin resistant and phleomycin sensitive transformants | Confirmed gene deletions |
| --- | --- | --- | --- | --- |
| H4-8A | No | 103 ± 7.4 | 24 ± 1 | 0 ± 0 |
| H4-8A | Yes | 125 ± 3.8 | 26 ± 2.1 | 7 ± 1.5 |
| H4-8A Δku80 | No | 4 ± 1.2 | 1 ± 0.6 | 0 ± 0 |
| H4-8A Δku80 | Yes | 120 ± 6.1 | 60 ± 1 | 30 ± 3.6 |

**Supplementary Table 2.** Confirmed gene deletions using repair templates with reduced homology arm lengths (see Figure 1). Values are normalized to the number of deletion strains per 2 x 10^7^ protoplasts. The average values and standard deviations are given.

| **Homology arm length** | **Confirmed gene deletions** |
| --- | --- |
| 1000 bp | 3.6 ± 1.9 |
| 750 bp | 2.8 ± 1.4 |
| 500 bp | 1.6 ± 0.5 |
| 250 bp | 2 ± 0 |
| 100 bp | 0.3 ± 0.4 |
